# Eating disorder symptoms among children and adolescents in Germany before and after the onset of the COVID-19 pandemic

**DOI:** 10.3389/fpsyt.2023.1157402

**Published:** 2023-05-26

**Authors:** Ann-Kathrin Napp, Anne Kaman, Michael Erhart, Joachim Westenhöfer, Ulrike Ravens-Sieberer

**Affiliations:** ^1^Department of Child and Adolescent Psychiatry, Psychotherapy, and Psychosomatics, University Medical Center Hamburg-Eppendorf, Hamburg, Germany; ^2^Department of Health Sciences, Faculty of Life Sciences, Competence Center Health, Hamburg University of Applied Sciences, Hamburg, Germany; ^3^Department of Public Health, Alice Salomon University of Applied Sciences, Berlin, Germany; ^4^Department of Psychology, Apollon University of Applied Sciences, Bremen, Germany

**Keywords:** eating disorder symptoms, disordered eating, youth, mental health, SARS-CoV-2, SCOFF

## Abstract

**Background:**

Disordered eating is highly prevalent among children and adolescents. Since the outbreak of the COVID-19 pandemic, hospitalizations due to eating disorders have peaked and overweight has risen. The aim of this study was to determine differences in the prevalence of eating disorder symptoms among children and adolescents in Germany before and after the onset of the COVID-19 pandemic and to identify associated factors.

**Materials and methods:**

Eating disorder symptoms and associated factors were examined in a sample of *n* = 1,001 participants of the nationwide population-based COPSY study in autumn 2021. Standardized and validated instruments were used to survey 11–17-year-olds along with a respective parent. To identify differences in prevalence rates, logistic regression was used to compare results with data from *n* = 997 participants of the prepandemic BELLA study. Multiple logistic regression analyses were performed to examine associations with relevant factors in the pandemic COPSY sample.

**Results:**

Eating disorder symptoms were reported by 17.18% of females and 15.08% of males in the COPSY study. Prevalence rates were lower overall in the COPSY sample compared to before the pandemic. Male gender, anxiety, and depressive symptoms were associated with increased odds for eating disorder symptoms in the pandemic.

**Conclusion:**

The pandemic underscores the importance of further research, but also prevention and intervention programs that address disordered eating in children and adolescents, with a focus on age - and gender-specific differences and developments. In addition, screening instruments for eating disorder symptoms in youths need to be adapted and validated.

## Introduction

For more than 2 years, the daily lives of people around the world have been affected by the outbreak of the SARS-CoV-2 pandemic. Although children and adolescents experience fewer symptoms of a COVID-19 infection compared to adults (1), the pandemic has severely impaired their social, school and family life and poses a great challenge to their mental health (2).

A growing body of evidence, including systematic reviews and longitudinal studies at international (3, 4) and national level (5, 6), reports an increase in a range of mental health problems, such as depression and anxiety symptoms, as well as lower quality of life. Given that mental health problems in childhood are associated with an enhanced risk for mental disorders in adulthood, these findings are of great public health importance (7).

One aspect of mental health that has been affected by the pandemic is eating behavior. Studies report a number of changes in eating behaviors during the pandemic, including an increase in restrictive eating, but also binge eating. Children consumed more salty and sweet snacks and were less physically active (8–10). Research findings also indicated an increase in weight among children and adolescents as well as the rise of overweight and obesity (11, 12).

Already at the onset of the pandemic, experts raised concerns about a potential increase in eating disorders (EDs) due to the loss of protective factors and elevated risk factors, such as disruption of routines (13–16). Even before the pandemic, studies have reported an increase in prevalence and incidence rates of EDs over time across ages and genders (17, 18). EDs are associated with increased mortality rates, comorbidity, and long-term functional impairments, including chronicity (19–22). An early age of onset is related to longer duration of illness and higher symptomatology (23). Prior to the pandemic, symptoms of EDs, like a distorted body image and restrictive eating, were found in approximately 20% of German adolescents (24). However, it is still unclear to what extent the COVID-19 pandemic has impacted ED symptoms in children and adolescents. Considering that these symptoms are precursors to the development of EDs (25–27), research about ED symptoms in youth is crucial to identify at-risk groups.

In the etiology and course of disordered eating behaviors, a number of individual, family, societal, and environmental factors play a role, in addition to sociodemographic factors such as female gender and migration background (24, 28, 29). Thus, self-efficacy, family climate, and social support have been identified as protective factors (28, 30–33). Disordered eating behaviors have also been shown to be predicted by comorbid mental health problems such as depression and anxiety (28, 34, 35). Also, a higher level of emotional problems and parental depression were identified as risk factors in children and adolescents (24, 28).

Recent studies have identified a range of potential contributing factors to EDs and disordered eating behavior, which are associated with the COVID-19 pandemic, including increased exposure to triggering social media content (36–38). Further, high COVID-19-related stress likely exacerbates pre-existing EDs and puts individuals at higher risk for ED symptoms such as binge eating, restrictive dieting, and body image concerns (39–41). Pandemic-related contact restrictions increased feelings of loneliness (42), a feeling closely related to EDs (43–45). At the same time, family conflicts escalate more frequently during the pandemic (46). Findings from two systematic reviews show that family conflicts were associated with worse ED outcomes among adolescents (37, 47).

A growing number of systematic reviews addressing EDs and disordered eating behaviors in the pandemic emphasize that most existing studies focus on clinical samples with a history of EDs (40, 47–49). Despite the early age of onset of EDs and their high prevalence in adolescents (18, 50), there are few studies focusing on these vulnerable populations since the onset of the pandemic. Adolescents with preexisting EDs appear to be at high risk for recurrence, exacerbation of symptoms, and severe impairment (51–55). Incidence rates of EDs have also increased, particularly among adolescents with anorexia nervosa (56, 57). In line with these findings, clinicians report substantial increases in the symptom severity and hospitalizations of children and adolescents with EDs since the onset of the COVID-19 pandemic (43, 56, 58–61). An increase in hospital referrals related to diagnosed EDs was also found by analyzing health insurance records for children and adolescents in Germany (62).

Yet, it is unclear whether this rise in hospital admissions and incidences is due to an exacerbation of symptoms in groups already at risk or to an increase in disordered eating in the general population (16). Large-scale population-based studies are still scare and results of existing studies focusing on children and adolescents vary (36, 63, 64). Among adults, studies mostly report a worsening of ED symptoms, such as an increase in binge eating, restrictive dieting, and worries about food and figure (48). An overall increase in the prevalence of eating pathology between the pre-and peri-COVID-19 era from 15.3 to 23.3% was reported in a recent meta-analysis (65).

Considering that most population-based studies are based on cross-sectional study designs and retrospective recall and are of low or moderate methodological quality (37, 48), representative studies in general populations are needed to estimate the burden of ED symptoms in the pandemic (16, 66). Furthermore, there is a need to systematically assess changes in disordered eating behaviors that have arisen and to investigate which existing and new emerging risk factors might influence ED symptoms in the pandemic.

Building on findings prior to and during the COVID-19 pandemic, the present study aims to fill the aforementioned research gap by answering the following research questions:

1. What is the current prevalence of ED symptoms in children and adolescents in Germany?
2. How has this prevalence changed in the general population and in age - and gender-specific subgroups compared with prepandemic findings?
3. Which factors (general and pandemic-specific) are related to ED symptoms among children and adolescents in the pandemic?

Based on these findings, recommendations for further research and clinical practice are drawn. The study will further inform policy makers and professionals about the impact of the pandemic on disordered eating among children and adolescents in Germany.

## Materials and methods

### Study design and sample

The longitudinal, population-based COPSY study (COVID-19 and Psychological Health) investigates the impact of the COVID-19 pandemic on the mental health of children and adolescents in Germany. It has been conducted since the beginning of the COVID-19 pandemic in 2020. The first wave of the COPSY study took place in May/June 2020. During a nationwide lockdown in Germany, participants were re-contacted in winter 2020/2021 for the second wave of the COPSY study.

After a summer with low infection rates and loosened restrictions, *n* = 1,618 families with children aged 7 to 18 years agreed to participate in the third wave of the COPSY study and completed the online survey between September and October 2021. A cross-sectional subsample of *n* = 1,001 children and adolescents aged 11 to 17 years who participated in the third wave of the COPSY study and provided information on eating disorder symptoms was included in the present analysis. The method and design of the COPSY study were developed in accordance with the population-based BELLA study, which is the mental health module of the National Health Survey of Children and Adolescents in Germany (KiGGS) (67). Data from *n* = 977 participants of the second wave of the KiGGS and the parallel fourth wave of the BELLA study (2014–2017) were used as a reference sample prior to the pandemic.

The datasets of the COPSY and BELLA study were each weighted to reflect the sociodemographic characteristics of the German population. Weights for the COPSY sample were calculated according to the 2018 Microcensus. Individual weights ranged from 0.2 to 3.93. Further details about the study design and methodology of the COPSY and BELLA studies are provided elsewhere (5, 68).

The COPSY study was approved by the Local Psychological Ethics Committee (LPEK-0151) and the data protection commissioner of the University of Hamburg.

### Measures

#### Sociodemographic information

Children and adolescents self-reported their age and gender. Information on parental education, migration background and occupational status were obtained in the proxy survey among parents. Parental education status was classified according to the Comparative Analyses of Social Mobility in Industrial Nations (CASMIN) (69).

#### Eating disorder symptoms

ED symptoms were assessed using the SCOFF (Sick, Control, One stone, Fat, Food) screening instrument (70). The five dichotomous questions of the SCOFF address core features of anorexia nervosa and bulimia nervosa, including deliberate vomiting, loss of control over eating, distorted body image, impact of food on life and weight loss. The latter item was adapted, rewording the weight loss of one stone to six kilograms as it has been done in other studies (24, 71). The diagnostic accuracy of the SCOFF was considered to be good overall according to a meta-analysis of international studies (72). Results from German studies vary. While a validation study in a representative sample of German adults found low sensitivity and a high number of false negatives (73), overall satisfactory psychometric properties, but a low positive predictive value were found in a sample of 12-year-olds (74). In addition, low internal consistency (Cronbach’s *α* = 0.44–0.66) was found in most studies (75). The SCOFF is known to have a tendency toward overinclusion, which is why reaching the cut-off for ED symptoms does not necessarily imply having an eating disorder (76). Nevertheless, the SCOFF is considered a useful and effective screening tool for detecting symptoms of EDs (72, 74). An established cut-off score of ≥2 expresses suspicion of an ED and was applied.

#### Associated factors

Emotional problems were assessed by the parent-reported version of the respective subscale of the Strengths and Difficulties Questionnaire (SDQ) (77). Participants self-reported symptoms of depression using seven items from the German version of the Center for Epidemiological Studies Depression Scale (CES-DC) (78, 79) and anxiety on nine items of the subscale generalized anxiety of the Screen for Child Anxiety Related Disorders (SCARED) (80). Parental depressive symptoms were assessed using the Patient Health Questionnaire (PHQ-8) (81). Scores can range between 0 and 10 for the SDQ, 0 and 21 for CES-DC, 0 and 18 for SCARED and 0 and 24 for PHQ-8. For all scales, higher scores indicate stronger symptoms.

A four item-subscale of the Family Climate Scale (FCS) was administered to assess family cohesion (82). Social support was self-reported using four modified items from the Social Support Scale (SSS) (83, 84). Sum scores range between 4 and 16 for the FCS and 4 and 20 for SSS. The 5-item Personal Resources Scale (PRS) was administered to assess self-efficacy with scores between 5 and 20 (85). Higher sum scores on FCS, SSS, and PRS correspond to more pronounced resources.

#### Pandemic specific factors

Children and adolescents rated feelings of loneliness using a short version of the Los Angeles Loneliness Scale (UCLA) (86). The four items used in this study had already been used with adolescents in population-based surveys (87), and a slightly modified response scale (1 = *never* to 5 = *always*) was used, resulting in an overall score between 4 and 20, with higher scores representing more loneliness.

Pandemic-related burden, increases in family conflicts and digital media use were assessed by newly developed items. Participants were asked to compare the frequency of family conflicts and duration of digital media use to the prepandemic time on a 5-point-likert scale (1 = *much less* to 5 = *much more*). Both variables were dichotomized into 1 = increase (response options *more* and *much more*) and 0 = no increase in family conflicts/digital media use (response options *much less*, *slightly less* and *same*).

### Data analysis

Based on self-reported ED symptoms according to the SCOFF, the prevalence of ED symptoms in the pandemic was calculated with 95%-*CIs*, stratified by age and gender. *N* = 8 participants of the COPSY study who reported their gender as “other” were excluded from the calculation of prevalence rates. Differences in symptomatology between age groups (11–13-year-olds vs. 14–17-year-olds) and gender were examined by bivariate chi-square (*χ*^2^) statistics and logistic regression.

To evaluate differences in the prevalence of ED symptoms before and during the pandemic, cross-sectional data from the BELLA study (prepandemic, control group) and the COPSY study (pandemic, index group) were pooled. Sociodemographic characteristics of COPSY and BELLA participants and differences in response to single items were compared using bivariate tests (*χ*^2^ and independent *t*-test). Furthermore, a multiple logistic regression with study (COPSY/BELLA group), age, gender, and interaction between gender and age as predictors for the total SCOFF score and specific ED symptoms was conducted. In a second explorative step, interactions between study and age as well as study and gender were included in the regression model.

To further describe the association between selected general and pandemic-specific factors (predictors) and ED symptomatology (outcome), unadjusted and adjusted logistic regression analyses with stepwise inclusion of general and pandemic-related factors were conducted using the COPSY dataset. All adjusted regression models were controlled for age, gender and the interaction of gender and age.

All analyses were carried out in IBM SPSS, version 27 and a *p* value ≤0.05 was considered as an indicator for significant differences or effects. Effect sizes Cohen’s *d* (*d*), Pearson’s *r* (*r*) and Phi (ɸ) are interpreted with regards to Cohen (88). Internal consistency was determined by Cronbach’s alpha (α) (89).

A power analysis was conducted prior to data analysis using the software G-Power 3.1. For determining the assumed OR to test for small effects in logistic regression analysis between two groups at a particular age (11–13 years, 14–17 years) and gender (girls, boys), we assumed an OR of 1.436/0.696 as suggested by Chinn (90). This resulted in a minimum required sample size to test for statistical significance with *p* (alpha) < 0.05 and a power of *p =* 0.8 of *n* = 302.

## Results

### Sample description

The present analysis is based on two subsamples of *n* = 1,001 (COPSY) and *n* = 997 (BELLA) 11-to 17-year-olds and a respective parent who participated in the COPSY or BELLA study. Girls participated slightly more often than boys in both studies (COPSY: 51.95%, BELLA: 54.16%). The mean age of children was 14.47 years (SD = 2.05) and 45.48 years (SD = 7.09) for participating parents in the COPSY study. Comparing the unweighted subsamples, participating children and adolescents and their parents in the COPSY study were about 1 year older than in the prepandemic BELLA subsample [Children’s age: *t*(1980.92) = 11.36, *p* < 0.001, *d* = 0.51; Parental age: *t*(1836.66) = 2.69, *p* = 0.007, *d* = 0.12]. Accordingly, differences were also found in children’s occupation with more children still attending school in BELLA [*χ*^2^ (1) = 90.70, *p* < 0.001, ɸ = −0.22]. Another significant difference was found in the educational level of parents [*χ*^2^ (2) = 81.83, *p* < 0.001, ɸ = 0.20], indicating that more parents reported a low educational level in the pandemic sample. Samples differed significantly in terms of migration background [*χ*^2^ (1) = 9.93, *p* = 0.002, ɸ = −0.07], with more migrants in the COPSY sample. With the exception of children’s age, the differences found were of small effect size. Sample details are provided in Table 1.

**Table 1:** Sociodemographic characteristics of the COPSY and BELLA subsamples used for the pooled analysis of eating disorder symptoms in children and adolescents before and after the onset of the COVID-19 pandemic.

	COPSY *n* = 1,001		BELLA *n* = 997	
	*n* (%)	*M* (SD)	*n* (%)	*M* (SD)
Gender				
Male	473 (47.25%)		457 (45.84%)	
Female	520 (51.95%)		540 (54.16%)	
Other	8 (0.80%)		n.A.	
Age (in years)		14.47 (2.05)		13.47 (1.87)
Occupation^1^				
School	860 (85.91%)		948 (97.73%)	
Other^2^	141 (14.09%)		22 (2.27%)	
Parental age (in years)^3^		45.48 (7.09)		44.72 (5.21)
Parental education^4,5^				
Low	166 (16.89%)		62 (6.30%)	
Medium	575 (58.49%)		536 (54.47%)	
High	242 (24.62%)		386 (39.23%)	
Migration background^6^				
Yes	175 (17.48%)		124 (12.45%)	
No	826 (82.52%)		872 (87.55%)	

Unweighted data; n.A., not assessed. ^1^BELLA: *n* = 27 missing. ^2^Other occupation includes apprenticeship, student or other. ^3^Deviating assessment in BELLA: Average age was used if both parents had participated in survey. ^4^Based on CASMIN classification. ^5^COPSY: *n* = 18 missing; BELLA: *n* = 13 missing. ^6^BELLA: *n* = 1 missing.

### Prevalence of eating disorder symptoms in the pandemic

The overall prevalence of ED symptoms in the analyzed sample according to SCOFF at a cut-off ≥2 was 16.20% (95% CI = 13.92-18.48%). Girls reported a higher but not significantly different prevalence compared to boys [17.18% (95% CI = 13.83-20.52%) vs. 15.08% (95% CI = 11.96–18.20%); *χ*^2^ (1) = 0.81, *p* = 0.369]. In terms of age, a higher prevalence was found in the older age group of 14-to 17-year-olds, where 17.12% (95% CI = 14.07-20.18%) reported disordered eating habits compared with 14.87% (95% CI = 11.45-18.28%) in 11-to 13-year-olds. However, this difference was not significant [*χ*^2^ (1) = 0.91, *p* = 0.34].

**Figure 1:**
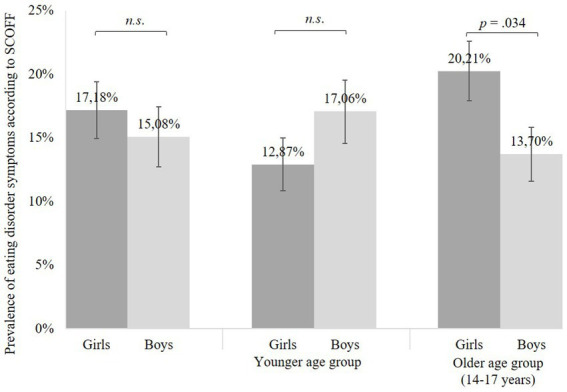
Prevalence of eating disorder symptoms in the COVID-19 pandemic by gender. Based on *n* = 993 participants of the COPSY study. *N* = 8 COPSY participants who reported other as gender are not included. Error bars indicate 95%-Confidence-Interval. Significant differences between groups were examined by chi-square tests. n.s., not significant.

Figure 1 shows the gender-specific prevalence of ED symptoms in different age groups. Among females, the older age group had a significantly higher prevalence of 20.21% (95% CI = 17.72-22.68%), while 12.87% (95% CI = 10.82-14.98%) of those under 14 years of age met the cut-off for ED symptomatology [*χ*^2^ (1) = 4.48, *p* = 0.034]. In the male subsample, an opposite trend, but no significant difference was observed. Thus, 17.06% (95% CI = 14.77-19.43%) of younger and 13.70% (95% CI = 11.57-15.83%) of older boys showed disordered eating behavior [*χ*^2^ (1) = 1.08, *p* = 0.299].

Contrasting trends were also observed within the younger and older subsamples. No significant difference was found between boys and girls in the younger age group [*χ*^2^ (1) = 1.42, *p* = 0.233], whereas older girls were significantly more likely to report disordered eating behaviors than boys [*χ*^2^ (1) = 4.36, *p* = 0.037]. A multiple logistic regression model with age, female gender, and interaction of age and female gender as predictors of eating disorder symptoms however was not significant [*χ*^2^ (3) = 5.71, *p* = 0.127].

Cronbach’s alpha for the SCOFF in this study was poor with *α* = 0.52 for the combined sample (*α* = 0.58 COPSY; *α* = 0.45 BELLA).

### Comparison with prepandemic findings from the BELLA study

Table 2 shows the prevalence of ED symptomatology in BELLA and COPSY across age groups and genders and the results of unadjusted logistic regressions. Descriptive analyses revealed a 3.9 percent point lower prevalence of eating disorder symptoms in COPSY compared to prepandemic results. This was confirmed by the regression analysis, as the odds for disordered eating were significantly lower in the COPSY study. Compared to girls, an inverse development was found in boys, who reported a significantly higher prevalence of 15.08% (95% CI = 11.96-18.20%) in the pandemic compared to 9.35% (95% CI = 6.78-11.92%) in the BELLA study prior to the pandemic. Unadjusted logistic regressions stratified by age group and gender showed that participation in the COPSY study was significantly associated with lower odds of ED symptoms in 14-to 17-year-olds (OR = 0.59; *p* < 0.001) and females (OR = 0.45; *p* < 0.001) but increased odds in boys (OR = 1.75; *p* = 0.004).

**Table 2:** Prevalence of eating disorder symptoms according to SCOFF in the prepandemic BELLA and the pandemic COPSY study, stratified by age group and gender.

	BELLA^a^ % [CI]	COPSY % [CI]	OR [CI]
Age			
11–13 years	12.56 [9.37–15.75]	14.87 [11.45–18.28]	1.23 [0.83–1.83]
14–17 years	**25.94 [22.22–29.66]**	**17.10 [14.07–20.18]**	0.59 [0.44–0.79]***
Gender			
Female	**31.72 [27.44–36.00]**	**17.18 [13.83–20.52]**	0.45 [0.33–0.61]***
Male	**9.35 [6.78–11.92]**	**15.08 [11.96–18.2]**	1.75 [1.18–2.58]**
Total	**20.10 [17.55–22.65]**	**16.20 [13.92–18.48]**	0.77 [0.61–0.97]*

Prevalence estimates are based on weighted data. BELLA: *n* = 977; COPSY *n* = 993. Significant differences (*p* < 0.05) were examined by unadjusted logistic regressions and are indicated in boldface. CI, 95%-Confidence Interval, OR, Odds Ratio. Discrepancies to reported prevalence for KiGGS 2 (24) resulting from smaller subsample. ^a^Reference category. *** *p* ≤ 0.001; **p ≤ 0.01; *p < 0.05.

In a multiple logistic regression model with age, female gender, and the interaction between the two as covariates, COPSY participants exhibited lower odds of disordered eating. Thus, participation in COPSY was associated with significantly reduced odds [OR 0.74 (95% CI = 0.58–0.94)] of ED symptoms. Age and the interaction of age and male gender were also significant predictors, with overall higher odds in females with increasing age. However, boys were less likely to show disordered eating with increasing age.

**Figure 2:**
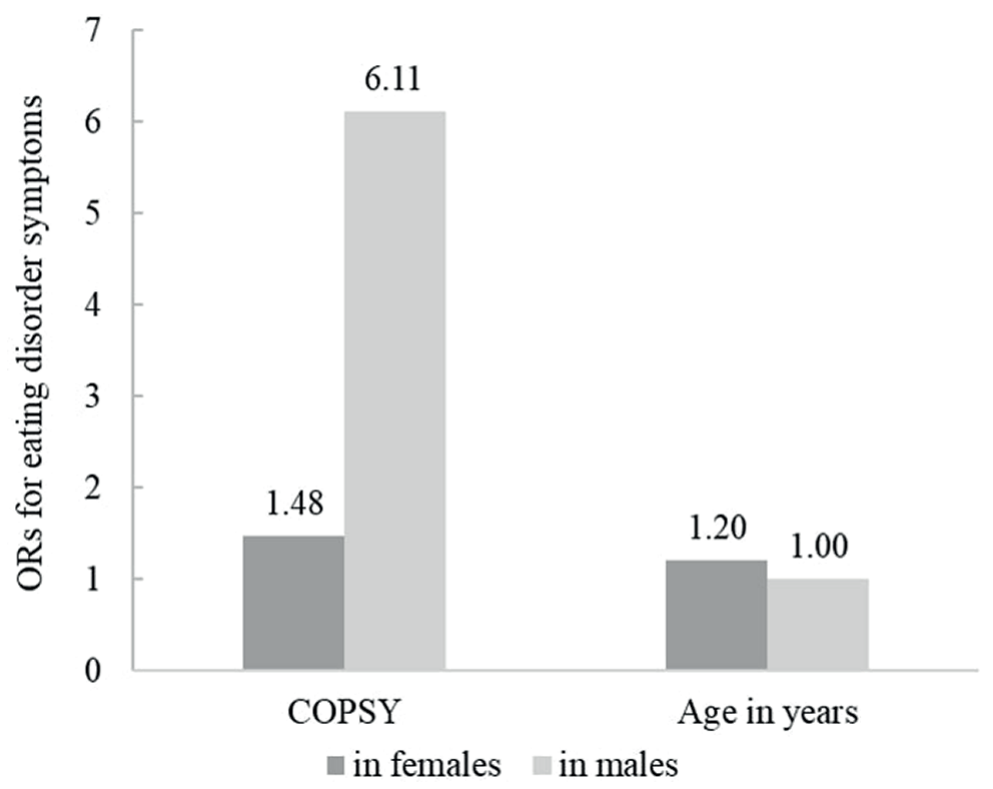
Visualization of interactions for eating disorder symptoms predictors. *n* = 1956, Outcome: eating disorder symptoms according to SCOFF; only significant interaction effects (*p* < 0.05) from model 2, Table 3 are included. OR, Odds Ratio.

Inclusion of interaction effects between study and age as well as study and gender improved the overall model fit according to Nagelkerke *R^2^.* Model 2 showed a significant interaction between study and male gender, indicating that boys were more likely to reach the cut off value of the SCOFF in the pandemic as compared to prior to the pandemic. In contrast, the main effects gender and study were not significant in model 2. Age remained a significant predictor. Details for models 1 and 2 are provided in Table 3. A visualization of significant interaction effects is provided in Figure 2.

**Table 3:** Results of stepwise multiple logistic regressions to predict eating disorder symptoms.

	Model 1			Model 2		
	*B* (SE)	OR [CI]	*p*	*B* (SE)	OR [CI]	*p*
Constant	**−2.65 (0.56)**	**0.07**	**<0.001**	**−3.31 (0.73)**	**0.04**	**<0.001**
Study^a^	**−0.30 (0.12)**	**0.74 [0.58; 0.94]**	**0.012**	0.39 (0.93)	1.48 [0.24; 9.08]	0.674
Age	**0.12 (0.04)**	**1.13 [1.04; 1.22]**	**0.003**	**0.18 (0.05)**	**1.20 [1.09; 1.33]**	**<0.001**
Male gender^b^	1.38 (0.90)	3.97 [0.68; 23.01]	0.125	1.00 (0.92)	2.72 [0.45; 16.46]	0.277
Age x Male gender	**−0.16 (0.06)**	**0.86 [0.76; 0.97]**	**0.014**	**−0.18 (0.07)**	**0.84 [0.74; 0.95]**	**0.005**
Study x Male gender				**1.42 (0.26)**	**4.14 [2.51; 6.83]**	**<0.001**
Study x Age				−0.09 (0.06)	0.92 [0.81; 1.04]	0.165
Model fit	*χ*^2^(4) = 61.87, *p* < 0.001 *Nagelkerke R^2^* = 0.05			*χ*^2^(6) = 97.82, *p* < 0.001 *Nagelkerke R^2^* = 0.08		

*n* = 1956, Outcome: eating disorder symptoms according to SCOFF; significant predictors (*p* < 0.05) are indicated in boldface. Reference category: ^a^BELLA study, ^b^Female gender. CI, 95% Confidence Interval; OR, Odds Ratio.

#### Symptom prevalence

Table 4 shows the prevalence for each of the five symptoms for ED assessed by the SCOFF in the prepandemic and pandemic sample. There were significant differences between the two samples for items 2 to 5, with fewer participants reporting symptoms of eating disorders in the COPSY study compared to BELLA. However, the proportion of participants reporting recent weight loss was almost twice as high in the pandemic. The highest prevalence was found for item 5, whereas intentional vomiting and recent weight loss were reported by less than 10% of participants in both samples.

**Table 4:** Symptom prevalence for specific eating disorder symptoms in the prepandemic BELLA and pandemic COPSY study.

SCOFF Items	BELLA % [CI]	COPSY % [CI]
Deliberate vomiting (Item 1)	3.59 [2.41–4.78]	5.09 [3.73–6.46]
Loss of control over eating (Item 2)	**19.83 [17.29–22.38]**	**11.99 [9.98–14.00]**
Recent weight loss (Item 3)	**3.90 [2.67–5.14]**	**7.29 [5.68–8.90]**
Distorted body image (Item 4)	**17.11 [14.70–19.52]**	**11.29 [9.33–13.25]**
Impact of food on life (Item 5)	**25.85 [23.05–28.65]**	**21.78 [19.22–23.34]**

Outcome: response frequency to specific SCOFF items. COPSY *n* = 1,001; BELLA *n* = 997, Missing: Item 1: *n* = 51, Item 2: *n* = 54; Item 3: *n* = 50; Item 4: *n* = 56; Item 5: *n* = 56. CI, 95% Confidence Interval. Significant differences (*p* < 0.05) were examined by chi-square test and are indicated in bold.

Multiple regressions for individual symptoms revealed that the interaction of study and male gender was associated with two-to fourfold increased odds for all symptoms [OR = 2.48 (item 4)–3.76 (item 2), *p* < 0.05]. As in the full model, the interaction of age and male gender was related to lower odds for items 1 to 4 [OR = 0.70 (item 1)–0.80 (item 3), *p* < 0.05], whereas the interaction of study and age was associated with lower odds for item 5 (OR = 0.77, *p* < 0.001). Higher age was associated with increased odds for items 2 to 4 [OR = 1.15 (item 4)–1.38 (item 3), *p* < 0.05]. The strongest effects were found for male gender as a predictor for items 1 and 2 [OR = 65.44, *p* = 0.011 (item 1); OR = 19.03, *p* = 0.002 (item 2)] and for participation in the COPSY study as a predictor for item 5 (OR = 19.95, *p* < 0.001). Results of the multiple logistic regression analysis for each of the five symptoms assessed by the SCOFF are provided in Supplementary Table S1.

### Associated factors

#### Intercorrelations of predictor variables

Correlations between general and pandemic-specific predictor and control variables are shown in Table 5. Most variables displayed small to medium correlations. Sociodemographic variables had only weak correlations, whereas moderate and strong correlations were found between other predictors. Symptoms of depression, anxiety, and emotional problems intercorrelated with large effects (*r* ≥ 0.60, *p* < 0.001), and showed the strongest correlations with other variables. Negative correlations were found between all non-sociodemographic variables and social support, personal resources, and family climate, which in turn correlated positively with each other at *r* ≥ 0.35. Among pandemic factors the strongest correlation was found between loneliness and depressive symptoms (*r* = 0.53, *p* < 0.001). Loneliness as well as increased family conflicts and digital media use showed significant but small associations with symptoms of mental health problems. Collinearity statistics indicated no multicollinearity (*VIF* 1.02–2.44, tolerance 0.43–0.98), according to Menard (91) and Myers (92).

**Table 5:** Intercorrelations of predictors for multiple logistic regression.

	1	2	3	4	5	6	7	8	9	10	11	12	13
1. Age	–												
2. Female gender^a^	0.01	–											
3. Migration background^a^	−0.01	0.04	–										
4. Emotional problems (SDQ)	−0.07*	0.15***	0.06	–									
5. Depressive symptoms (CED-DC)	0.05	0.09**	0.07*	0.67***	–								
6. Anxiety symptoms (SCARED)	0.01	0.13***	0.05	0.61***	0.60***	–							
7. Parental depressive symptoms (PHQ-8)	−0.04	−0.04	0.08*	0.44***	0.39***	0.30***	–						
8. Personal resources (PRS)	0.01	−0.05	−0.08*	−0.51***	−0.55***	−0.50***	−0.29***	–					
9. Social support (SSS)	0.09**	0.03	−0.06	−0.30***	−0.40***	−0.29***	−0.18***	0.35***	–				
10. Family climate (FCS)	−0.08*	0.01	−0.07*	−0.35***	−0.42***	−0.31***	−0.25***	0.47***	0.60***	–			
11. Pandemic burden^a^	−0.05	0.01	0.05	0.21***	0.21***	0.19***	0.18***	−0.26***	−0.10***	−0.10**	–		
12. Increased conflicts^a^	−0.09**	0.06	0.04	0.33***	0.31***	0.24***	0.29***	−0.24***	−0.25***	−0.37***	0.13***	–	
13. Loneliness (UCLA)	0.01	−0.01	0.08*	0.44***	0.53***	0.46***	0.30***	−0.45***	−0.34***	−0.32***	0.22***	0.25***	–
14. Increased digital media use^a^	−0.01	−0.02	0.09**	0.16***	0.20***	0.21***	0.15***	−0.14***	−0.05	−0.08*	0.15***	0.17***	0.25***

Pearson correlations for two continuous predictors (Pearson’s r), point-biserial for continuous and dichotomous predictors (r_pb_), chi-square for two dichotomous predictors (ɸ). ^a^Dichotomous predictor; *** *p* ≤ 0.001; **p ≤ 0.01; *p < 0.05.

CES-DC, Center for Epidemiological Studies Depression Scale; FCS, Family Climate Scale; PHQ-8, Patient Health Questionnaire; PRS, Personal Resources Scale; SCARED, Screen for Child Anxiety Related Disorders; SDQ, Strengths and Difficulties Questionnaire; SSS, Social Support Scale; UCLA, Los Angeles Loneliness Scale.

#### Results of the univariate logistic regression analyses

In a series of unadjusted logistic regressions, all factors, except for female gender and migration background were significantly associated with ED symptomatology in the pandemic. Thus, higher emotional problems, symptoms of depression and anxiety as well as higher parental depressive symptoms were associated with increased odds for disordered eating. In contrast, higher personal resources, social support, and a better family climate were associated with reduced odds. Further, all pandemic-specific factors (increased digital media use, family conflicts, higher burden and greater loneliness) were related to higher *ORs*. High pandemic burden and increased family conflicts were associated with a more than twice as high chance of disordered eating. The results of the univariate logistic regressions are provided in Supplementary Table S2.

#### Results of the multiple logistic regression analyses

The results of the multiple logistic regression are presented in Table 6 for the full sample and stratified by gender in Supplementary Table S3. In model 1, only general factors as predictors of ED symptomatology in the total sample were incorporated. Generalized anxiety, symptoms of depression, and gender were significantly associated with disordered eating. Inclusion of factors related to the pandemic (model 2) did not improve the model significantly. Female gender was associated with reduced odds (OR = 0.07; *p* = 0.044), while symptoms of anxiety (OR = 1.08; *p* = 0.004) and depression (OR = 1.10; *p* = 0.002) were associated with slightly increased odds of disordered eating. None of the other significant factors from the univariate model predicted eating disorder symptomatology in the multiple models.

**Table 6:** Multiple logistic regression to predict eating disorder symptoms.

	Model 1			Model 2		
	*B* (SE)	OR [CI]	*p*	*B* (SE)	OR [CI]	*p*
General factors						
Constant	−1.98 (1.30)	0.14	0.129	−2.40 (1.40)	0.09	0.085
Age	−0.08 (0.06)	0.92 [0.81–1.05]	0.200	−0.08 (0.07)	0.93 [0.81–1.05]	0.237
Gender^a^	**−2.55 (1.29)**	**0.08 [0.01–0.97]**	**0.048**	**−2.61 (1.30)**	**0.07 [0.01–0.93]**	**0.044**
Age x Gender	0.17 (0.09)	1.19 [0.99–1.41]	0.059	0.17 (0.09)	1.19 [0.997–1.42]	0.055
Migration background^b^	0.16 (0.23)	1.17 [0.74–1.84]	0.499	0.15 (0.23)	1.17 [0.74–1.84]	0.511
Anxiety symptoms (SCARED)	**0.07 (0.03)**	**1.08 [1.02–1.13]**	**0.004**	**0.08 (0.03)**	**1.08 [1.02–1.13]**	**0.004**
Depressive Symptoms (CES-DC)	**0.10 (0.03)**	**1.10 [1.04–1.17]**	**0.001**	**0.10 (0.03)**	**1.10 [1.04–1.17]**	**0.002**
Emotional problems (SDQ)	0.08 (0.05)	1.09 [0.98–1.21]	0.124	0.08 (0.06)	1.08 [0.97–1.20]	0.164
Parental depressive symptoms (PHQ-8)	0.01 (0.02)	1.01 [0.97–1.05]	0.736	0 (0.02)	1.00 [0.97–1.04]	0.876
Family climate (FCS)	−0.07 (0.05)	0.93 [0.85–1.02]	0.127	−0.06 (0.05)	0.94 [0.85–1.03]	0.205
Personal resources (PRS)	0.01 (0.04)	1.01 [0.93–1.09]	0.824	0.01 (0.04)	1.01 [0.93–1.10]	0.763
Social support (SSS)	0.02 (0.04)	1.02 [0.95–1.10]	0.599	0.02 (0.04)	1.02 [0.94–1.10]	0.681
Pandemic factors						
Increased digital media use^c^				0.1 (0.20)	1.11 [0.75–1.62]	0.605
Increased family conflicts^d^				0.2 (0.23)	1.22 [0.78–1.90]	0.380
Pandemic burden^e^				0.32 (0.30)	1.38 [0.76–2.50]	0.292
Loneliness (UCLA)				−0.01 (0.03)	0.99 [0.93–1.05]	0.666
Model fit	*χ*^2^(11) = 109.94, *p* < 0.001 *Nagelkerke R^2^* = 0.177			*χ*^2^(15) = 112.46, *p* < 0.001 *Nagelkerke R^2^* = 0.181		

*n* = 1,001; Outcome: eating disorder symptoms according to SCOFF; significant predictors (*p* < 0.05) are indicated in boldface. Reference category: ^a^Not female; ^b^No migration background; ^c^No increase in digital media use; ^d^No increase in conflicts; ^e^Not burdened by the pandemic.

CI, 95% Confidence Interval; CES-DC, Center for Epidemiological Studies Depression Scale; FCS, Family Climate Scale; OR, Odds Ratio; PHQ-8, Patient Health Questionnaire; PRS, Personal Resources Scale; SCARED, Screen for Child Anxiety Related Disorders; SDQ, Strengths and Difficulties Questionnaire; SSS, Social Support Scale; UCLA, Los Angeles Loneliness Scale.

Stratified regressions by sex (Supplementary Table S3) revealed that anxiety symptoms were a significant predictor only in female adolescents (OR = 1.09; *p* = 0.012), whereas depressive symptoms were associated with increased odds for eating disorder symptoms in both females (OR = 1.09; *p* = 0.028) and males (OR = 1.13; *p* = 0.020). All multiple logistic regression models were statistically significant with Nagelkerke *R^2^* ranging between 0.181 and 0.202.

## Discussion

The aim of this study was to estimate the prevalence of ED symptoms in children and adolescents 1.5 years after the outbreak of the COVID-19 pandemic in Germany and to compare the results with prepandemic data. In addition, factors associated with ED symptoms during the pandemic were to be identified.

### Prevalence of eating disorder symptoms in the pandemic

An overall prevalence of ED symptoms of 16.20% was found, with 17.18% of female and 15.08% of male participants reaching the SCOFF cut-off. Other studies administering the SCOFF in more homogenous samples reported considerably higher prevalence rates of 18.4 and 31.1% for males and 25.3 and 51.8% for females in 2020/2021, respectively (63, 93). According to our descriptive results, prevalence was slightly higher among girls and older participants. However, significant gender differences were only found in 14-to 17-year-olds. Most studies from the pandemic period and before report similar but more pronounced differences in disordered eating behaviors between genders and age groups (65, 71).

### Comparison with prepandemic findings

We found a significant difference in the prevalence of ED symptoms compared with the prepandemic BELLA study, with an overall reduced likelihood for ED symptoms in the pandemic. Prior to the pandemic, girls had a three times higher prevalence compared to boys. Interestingly, findings from our regression analysis indicate that in boys, risk for ED symptoms increased significantly in the pandemic, in contrast to girls. Consequently, boys had a higher prevalence during the pandemic compared to before it. As the effect found is based on a large standard error and CI, results should be interpreted with caution. Gender differences were found in terms of age-specific developments. Thus, boys were less likely to have ED symptoms with increasing age, whereas older girls were more likely to show disordered eating behavior.

While the symptoms “deliberate vomiting,” “loss of control over eating,” “distorted body image,” and “impact of food on life” decreased or did not differ significantly during the pandemic compared to the prepandemic sample, the percentage of participants reporting “recent weight loss” increased. In addition, boys were more likely to show all symptoms of EDs during the pandemic.

Contrary to our findings, a school-based study in Germany found no changes in disordered eating habits in the beginning of the pandemic compared to prepandemic data (64), while a significant increase of perceived disordered eating and overeating was observed in a sample of female adolescents in the summer/autumn 2021 (36). Also international studies using the SCOFF in older students found significant increases in the prevalence of ED symptoms in both male and female participants from 2018/2019 to the first and second year of the pandemic, respectively (63, 93).

Only limited evidence is available regarding the potential increase of ED symptoms in boys, particularly at a young age. Consistent with our findings, boys were more likely to show increased consumption of snacks, soft drinks, and carbohydrates and to gain weight during the pandemic, especially between the ages of 10 and 12 (8).The high prevalence of ED symptoms in boys may also be due to an increase in binge eating during the pandemic (9). This is underscored by evidence showing that subclinical forms of binge eating disorder were as common in boys as in girls even before the pandemic (94). In addition, the ongoing discussion concerning the historically female-oriented diagnostic framework and assessment of disordered eating should be considered (94, 95). An increase in diagnosed EDs was only found in young men between 20 and 24 years of age, but not boys in the first year of the pandemic according to German health insurance data (96). Others reported a decrease in the number of ED-related hospital admissions among boys, but an increase among girls (62). For girls, younger age was associated with increases in disordered eating and EDs (36, 97).

As noted above, in contrast to sharp increases in EDs, particularly anorexia nervosa, reported by clinicians and health care data, our findings show a decrease in disordered eating behaviors after the onset of the COVID-19 pandemic. There are several possible explanations for this discrepancy. First and foremost, ED symptoms do not necessarily lead to diagnosed EDs, so the number of reported diagnosed cases and self-reported prevalence may differ. This was also the case before the pandemic, and it has been suggested that this may be attributable to the awareness effect. Thus, greater societal awareness of ED and a greater willingness to seek medical consultation could explain the increase in diagnosed EDs (24, 98). The increase in clinically relevant cases could also be due to an exacerbation of symptoms in risk groups or patients with pre-existing EDs (e.g., 47, 54). As families had to stay at home during nationwide lockdowns, parents might have noticed disordered eating habits earlier and intervened. Further, given that family conflicts escalated more frequently during the pandemic, parents may have been more inclined to bring children in for treatment in order to reduce tensions at home.

Another hypothesis is that the pandemic has led to positive developments in children and adolescents with disordered eating behaviors. This might include families supporting at-risk children through supervised or shared mealtimes at home. Increased time for self-care and reflection may also be beneficial (37, 49). This is in line with the results of our univariate regression analysis, where family climate was identified as a protective factor.

Furthermore, the use of the SCOFF as an instrument to assess ED symptoms can be seen as a limitation. The low psychometric properties of the SCOFF such as a low positive predictive value and a high number of false negatives are particularly evident in heterogeneous population-based samples (73, 75). In line with others, we also found very low internal consistency (*α* = 0.52) (74). Given the limited reliability in this study, all observations need to be interpreted carefully. In addition, the SCOFF does not assess all major symptoms of disordered eating behaviors, including laxative abuse and excessive exercise. As a result, it does not depict symptoms of other highly prevalent eating disorders, such as binge eating disorder or newly emerging forms of disordered eating such as orthorexia nervosa (75, 99). However, the SCOFF was developed to detect core symptoms of anorexia nervosa and bulimia nervosa (70). Yet, studies show that the SCOFF captures more symptoms in overweight children and adolescents suggesting that, for example, item 2 (“*Do you worry you have lost control over how much you eat?*”) could be understood as experiencing binge eating (71). Because of their usability and efficiency, screening tools such as the SCOFF are essential for both clinical assessment and public health research to estimate the burden of EDs and to identify at-risk groups. As to date there is a lack of evaluated, standardized screening tools to measure EDs in children and adolescents (75, 100), there is high need for further research.

In addition, the time of data collection in the COPSY study should be taken into account. Since ED symptoms were first assessed 1.5 years after the onset of the pandemic in the third wave of the COPSY study, the prevalence at the beginning of the pandemic is unknown. Therefore, the progression of ED symptoms from the beginning to later points in the pandemic cannot be compared in the same way that other changes to mental health can. For instance, anxiety symptoms increased in the first year of the pandemic but decreased slightly in the third wave of the COPSY study (5). This might be due to greater awareness of the adverse impact of the pandemic on young people’s mental health and the increased availability of support services. To better understand the development of disordered eating behavior over the course of the pandemic and beyond, there is a high need for longitudinal studies.

### Associated factors

The results of the univariate regression analyses of the COPSY study showed that there were associations between all factors examined and a positive SCOFF score, with the exception of gender and migration background. However, a multiple regression model showed that only gender, depression and anxiety symptoms were associated with ED symptoms 1.5 years after the onset of the pandemic. The association between symptoms of anxiety and depression is consistent with findings from other studies conducted before and during the pandemic (34, 35, 101). However, in contrast to recent findings among adults (102), anxiety was only a significant predictor among girls. One possible explanation is that female gender has been identified as a risk factor for anxiety symptoms in the pandemic (103). Furthermore, we found that girls were less likely than boys to show ED symptoms 1.5 years after the pandemic outbreak when other factors were considered. As mentioned before, this contrasts with the reported increase in ED-related hospitalizations among girls (62).

In the first model, Nagelkerke *R^2^* was <0.2 and the addition of pandemic factors did not significantly improve model fit. Thus, none of the factors were significant in the multivariate model. This might be due to the fact that these factors become less significant in interaction with other factors. Furthermore, it is known that in addition to the investigated factors, there are other determinants for disordered eating. Besides predisposing factors like genetics, ethnicity, self-esteem and negative childhood experiences, these include stress factors like thin body preoccupation, negative life events, negative family perception and social pressure (30, 31, 104). Other factors that may be relevant in times of the pandemic could be uncertainty intolerance, food insecurity, and socioeconomic status (36, 47). The latter has been identified as a risk factor for higher weight gain (8) and other mental health problems in the pandemic (5).

### Strengths and limitations

This study has the following strengths. The COPSY study is one of the first nationwide population-based studies focusing on child and adolescent mental health following the COVID-19 pandemic outbreak (6). By comparing the results with nationally representative prepandemic data, it is possible to draw conclusions about changes in prevalence in specific subgroups. In addition, established instruments for the assessment of mental health as well as risk and protective factors were administered. This allowed the inclusion of a range of potential predictors in the analyses.

In addition to the use of the SCOFF despite its low psychometric properties, there are a number of other limitations. First, height and weight were not assessed in the COPSY study. Given the high prevalence of ED symptoms in overweight and underweight individuals (24, 71, 101) and the increase in overweight that has been reported in the pandemic (11), it is highly relevant to consider the association of body mass index with ED symptoms in the pandemic. Second, it is not possible to draw causal relationships between the reported associations given the cross-sectional design of the study. Third, it should be considered that biases are likely to occur in self-reported surveys. Since especially patients in the early stages of an ED often deny symptoms (105), this should be given particular consideration. Further, most pandemic-specific factors in the regression model were assessed with single items because of the broad range of issues covered by the COPSY study. Future studies should examine potential pandemic-specific risk factors in more detail by assessing them with standardized and validated instruments. Lastly, all findings of the COPSY study are not generalizable to other countries, especially given differences in the course and handling of the COVID-19 pandemic.

### Implications for further research and practice

To the best of our knowledge, this study provides the first estimate for the prevalence of self-reported ED symptoms among children and adolescents in a nationwide sample in Germany since the onset of the COVID-19 pandemic. Our findings indicate an overall decrease compared to prepandemic findings and highlight gender-specific developments. Thus, we found an increase of disordered eating habits among boys, especially in the younger age group. This emphasizes the need for further research, examining the relevance of gender- and age-specific developments of disordered eating in children and adolescents in the pandemic. In addition, family-based intervention and prevention programs targeting at-risk groups and taking up gender-and age-specific approaches are highly warranted.

Our results indicate that symptoms of anxiety and depression are significant predictors for ED symptoms in children and adolescents in the pandemic. Given that these have also increased in the pandemic (4), their association with ED symptoms needs to be examined in further studies to detect cause and effect relationships. In clinical practice, screening for ED symptoms to ensure early detection could also become part of the diagnosis and treatment of children and adolescents with depressive and anxiety symptoms. Furthermore, future research should focus on predictors of specific forms of EDs, such as anorexia nervosa, bulimia nervosa and binge eating disorder in the pandemic.

High-quality screening instruments are essential for the early detection of ED symptoms to prevent these symptoms from developing into clinical forms of EDs. By using valid and reliable screening instruments in longitudinal and large-scale population-based studies, it is possible to provide highly relevant and valid data to investigate the public health burden and incidence of ED symptoms in children and adolescents. Considering that adolescence is a high-risk period for the onset of EDs and that the pandemic has exacerbated mental health problems in young people, there is a high need to address better evaluation of existing instruments and to develop alternative screening tools that also allow for disease-specific screening in children and adolescents.

## Data availability statement

The raw data supporting the conclusions of this article will be made available by the authors, without undue reservation.

## Ethics statement

The studies involving human participants were reviewed and approved by Local Psychological Ethics Committee and the Commissioner for Data Protection of the University of Hamburg, Germany. Written informed consent to participate in this study was provided by the participants’ legal guardian/next of kin.

## Author contributions

A-KN performed the statistical analyses, interpreted the data, and wrote the first draft of the manuscript. UR-S and AK were principle investigators of the COPSY study, responsible for its design, funding, general decisions of measurement, supervised data cleaning and preparation, and revised the manuscript critically. JW and ME revised the manuscript critically. All authors contributed to the article and approved the final manuscript.

## Funding

The COPSY study was funded by the Kroschke Child Foundation, the Fritz and Hildegard Berg Foundation, the Jaekel Foundation and the Foundation “Wissenschaft in Hamburg.” The funders had no role in study design, data collection and analysis, decision to publish, or the preparation of the manuscript. We acknowledge financial support from the Open Access Publication Fund of UKE - Universitätsklinikum Hamburg-Eppendorfand DFG – German Research Foundation.

## Conflict of interest

The authors declare that the research was conducted in the absence of any commercial or financial relationships that could be construed as a potential conflict of interest.

## Publisher’s note

All claims expressed in this article are solely those of the authors and do not necessarily represent those of their affiliated organizations, or those of the publisher, the editors and the reviewers. Any product that may be evaluated in this article, or claim that may be made by its manufacturer, is not guaranteed or endorsed by the publisher.

## Abbreviations

CES-DC: Center for Epidemiological Studies Depression Scale
ED: Eating Disorder
FCS: Family Climate Scale
PHQ-8: Patient Health Questionnaire
PRS: Personal Resources Scale
SCARED: Screen for Child Anxiety Related Disorders
SCOFF: Sick, Control, One Stone, Fat, Food–Screening Instrument for eating disorder symptoms
SDQ: Strengths and Difficulties Questionnaire
SSS: Social Support Scale
UCLA: Los Angeles Loneliness Scale
CASMIN: Comparative Analyses of Social Mobility in Industrial Nations
COPSY: COVID-19 and Psychological Health.
