# Supplementary material for: Eating disorder symptoms among children and adolescents in Germany before and after the onset of the COVID-19 pandemic
Source: Front Psychiatry. 2023 May 26;14:1157402. doi: 10.3389/fpsyt.2023.1157402 (PMC10254422; doi:10.3389/fpsyt.2023.1157402)
Supplement: Supplementary file 1 [file Table_1.DOCX]

***Supplementary Material***

**Supplementary Table 1.** Results of multiple logistic regressions to predict each symptom of disordered eating.

|  | Item 1  (*n* = 1964) | | | Item 2  (*n* = 1962) | | | Item 3  (*n* = 1960) | | | Item 4  (*n* = 1958) | | | | Item 5  (*n* = 1953) | | | |  |
| --- | --- | --- | --- | --- | --- | --- | --- | --- | --- | --- | --- | --- | --- | --- | --- | --- | --- | --- |
|  | *B(SE)* | *OR [CI]* | *p* | *B* *(SE)* | *OR [CI]* | *p* | *B* *(SE)* | *OR [CI]* | *p* | | *B* *(SE)* | *OR [CI]* | *p* | | *B* *(SE)* | *OR [CI]* | *p* | |
| Constant | **-4.84 (1.51)** | **0.01** | **.001** | **-2.99 (0.74)** | **0.05** | **<.001** | **-7.51 (1.54)** | **0** | **<.001** | | **-2.93 (0.77)** | **0.05** | **<.001** | | **-4.35 (0.71)** | **0.01** | **<.001** | |
| Study^a^ | -0.75 (1.84) | 0.47  [0.01; 17.19] | .682 | -0.89 (1.00) | 0.41  [0.06; 2.91] | .372 | 1.44 (1.75) | 4.23  [0.14; 130.37] | .410 | | -0.26 (1.01) | 0.77  [0.11; 5.59] | .794 | | **2.99 (0.83)** | **19.95**  **[3.95; 100.73]** | **<.001** | |
| Age | 0.13 (0.11) | 1.14  [0.93; 1.41] | .205 | **0.15 (0.05)** | **1.17**  **[1.05; 1.29]** | **.003** | **0.32 (0.1)** | **1.38**  **[1.13; 1.69]** | **.002** | | **0.14 (0.05)** | **1.15**  **[1.03; 1.28]** | **.011** | | **0.26 (0.05)** | **1.30**  **[1.18; 1.43]** | **<.001** | |
| Male gender^b^ | **4.18 (1.65)** | **65.44**  **[2.58; 1662.73]** | **.011** | **2.95 (0.97)** | **19.02**  **[2.84; 127.26]** | **.002** | 2.47 (1.56) | 11.81  [0.56; 250.3] | .113 | | 1.81 (1.03) | 6.10  [0.82; 45.54] | .078 | | -0.89 (0.82) | 0.41  [0.08; 2.05] | .278 | |
| Age x Male gender | **-0.36 (0.12)** | **0.70**  **[0.55; 0.88]** | **.003** | **-0.31 (0.07)** | **0.73**  **[0.64; 0.84]** | **<.001** | **-0.22 (0.11)** | **0.80**  **[0.65; 0.98]** | **.034** | | **-0.23 (0.07)** | **0.79**  **[0.69; 0.92]** | **.002** | | 0.01 (0.06) | 1.01  [0.90; 1.13] | .909 | |
| Study x Male gender | **1.19 (0.5)** | **3.27**  **[1.23; 8.68]** | **.017** | **1.32 (0.28)** | **3.76**  **[2.19; 6.44]** | **<.001** | **1.06 (0.44)** | **2.87**  **[1.21; 6.84]** | **.017** | | **0.91 (0.29)** | **2.48**  **[1.41; 4.36]** | **.002** | | **1.04 (0.22)** | **2.82**  **[1.84; 4.34]** | **<.001** | |
| Study x Age | 0.04 (0.13) | 1.04  [0.82; 1.33] | .752 | -0.02 (0.07) | 0.98  [0.86; 1.12] | .767 | -0.09 (0.12) | 0.91  [0.73; 1.15] | .428 | | -0.04 (0.07) | 0.96  [0.84; 1.01] | .549 | | **-0.27 (0.06)** | **0.77 [0.68; 0.86]** | **<.001** | |
| Model fit | χ² (6) = 17.22, p = .008  *Nagelkerke R²* = .03 | | | χ² (6) = 103.36, p < .001  *Nagelkerke R²* = .09 | | | χ² (6) = 31.12, p < .001  *Nagelkerke R²* = .05 | | | χ² (6) = 90.88, p < .001  *Nagelkerke R²* = .08 | | | | χ² (6) = 71.56, p < .001  *Nagelkerke R²* = .05 | | | |  |

*Notes.* Outcome: Eating disorder symptomatology according to SCOFF. Significant predictors (*p* <.05) are indicated in boldface. Reference category: ^a^ Participation in pre-pandemic BELLA study, ^b^ Female gender. *CI* = 95% Confidence Interval, *OR* = Odds Ratio.

|  | | **Model fit**  Nagelkerke *R²* | ***B (SE)*** | ***OR [CI]*** | ***p*** | |
| --- | --- | --- | --- | --- | --- | --- |
| Sociodemographic factors | | | | | |  |
| Female gender^a^ | | χ² (1)=0.70; *p* = .401  *R²* = .001 | 0.14 (0.17) | 1.2 [0.83-1.62] | .402 | |
| Migration background^b^ | | χ² (1)=1.71; *p* = .191  *R²* = .003 | 0.28 (0.21) | 1.77 [0.88-2.02] | .183 | |
| General factors |  | | | | |  |
| Anxiety symptoms (SCARED) | | **χ² (1)= 72.35; *p* < .001**  ***R²* = .119** | **0.16 (0.02)** | **1.17 [1.13-1.22]** | **<.001** | |
| Depressive symptoms (CES-DC) | | **χ² (1)= 83.92; *p* < .001**  ***R²* = .137** | **0.18 (0.02)** | **1.2 [1.15-1.24]** | **<.001** | |
| Emotional problems (SDQ) | | **χ² (1)=71.11; *p* < .001**  ***R²* = .117** | **0.3 (0.04)** | **1.35 [1.26-1.45]** | **<.001** | |
| Parental depressive symptoms (PHQ-8) | | **χ² (1)= 22.31; *p* < .001**  ***R²* = .037** | **0.18 (0.02)** | **1.08 [1.05-1.11]** | **<.001** | |
| Family climate (FCS) | | **χ² (1)=31.93; *p* < .001**  ***R²* = .053** | **-0.19 (0.03)** | **0.83 [0.77-0.88]** | **<.001** | |
| Personal resources (PRS) | | **χ² (1)=41.13; *p* < .001**  ***R²* = .068** | **-0.19 (0.03)** | **0.83 [0.78-0.88]** | **<.001** | |
| Social support (SSS) | | **χ² (1)=17.94; *p* < .001**  ***R²* = .03** | **-0.12 (0.03)** | **0.88 [0.84-0.94]** | **<.001** | |
| Pandemic specific factors |  | | | | |  |
| Increased digital media use^c^ | | **χ² (1)= 7.25; *p* = .007**  ***R²* = .012** | **0.47 (0.17)** | **1.59 [1.13-2.24]** | **.008** | |
| Increased family conflicts^d^ | | **χ² (1)= 21.28 *p* < .001**  ***R²* = .036** | **0.87 (0.18)** | **2.39 [1.66-3.42]** | **<.001** | |
| Pandemic burden^e^ | | **χ² (1)= 9.91; *p* = .002**  ***R²* = .017** | **0.82 (0.28)** | **2.27 [1.3-3.95]** | **.004** | |
| Loneliness (UCLA) | | **χ² (1)= 31.04; *p* < .001**  ***R²* = .052** | **0.13 (0.02)** | **1.14 [1.09-1.19]** | **<.001** | |

**Supplementary Table 2**. Results of univariate logistic regressions. Predictors for eating disorder symptoms among children and adolescents in the COVID-19 pandemic.

*Notes. n* = 1,001; Outcome: Eating disorders symptoms according to SCOFF. Reference category: ^a^ Not female; ^b^ No migration background; ^c^ No increase in digital media use; ^d^ No increase in conflicts; ^e^ Not burdened by the pandemic. Abbreviations: *CI* = 95% Confidence Interval; CES-DC = Center for Epidemiological Studies Depression Scale; FCS = Family Climate Scale; *OR* = Odds Ratio; PHQ-8 = Patient Health Questionnaire; PRS = Personal Resources Scale; SCARED = Screen for Child Anxiety Related Disorders; SDQ = Strengths and Difficulties Questionnaire; SSS = Social Support Scale; UCLA = Los Angeles Loneliness Scale.

**Supplementary Table 3.** Multivariate logistic regression to predict eating disorder symptoms, stratified by gender.

|  | Model 1 | | | | | | Model 2 | | | | | | |
| --- | --- | --- | --- | --- | --- | --- | --- | --- | --- | --- | --- | --- | --- |
|  | Females (*n* = 520) | | | Males (*n* = 473) | | | Females (*n* = 520) | | | Males (*n* = 473) | | | |
|  | *B (SE)* | *OR*  *[CI]* | *p* | *B (SE)* | *OR*  *[CI]* | *p* | *B (SE)* | *OR*  *[CI]* | *p* | *B (SE)* | *OR*  *[CI]* | *p* |  |
| **General factors** |  |  |  |  |  |  |  |  |  |  |  |  |  |
| Constant | **-4.58 (1.72)** | **0.01** | **.008** | -1.93 (1.63) | 0.14 | .235 | **-5.25 (1.85)** | **0.01** | **.005** | -2.41 (1.81) | 0.09 | .183 |  |
| Age | 0.09 (0.06) | 1.09  [0.96-1.24] | .169 | -0.08 (0.07) | 0.93  [0.81-1.06] | .261 | 0.12 (0.07) | 1.12  [0.99-1.28] | .074 | -0.08 (0.07) | 0.92  [0.81-1.06] | .253 |  |
| Migration background^b^ | 0.33 (0.31) | 1.39  [0.75-2.55] | .293 | -0.09 (0.35) | 0.91  [0.46-1.82] | .790 | 0.33 (0.32) | 1.39  [0.75-2.59] | .293 | -0.12 (0.36) | 0.89  [0.44-1.79] | .740 |  |
| Anxiety symptoms (SCARED) | **0.08 (0.03)** | **1.09**  **[1.01-1.16]** | **.018** | 0.05 (0.04) | 1.05  [0.97-1.13] | .225 | **0.09 (0.04)** | **1.09**  **[1.02-1.17]** | **.012** | 0.04 (0.04) | 1.04  [0.96-1.13] | .312 |  |
| Depressive Symptoms (CES-DC) | **0.08 (0.04)** | **1.08**  **[1.00-1.17]** | **.037** | **0.13 (0.05)** | **1.14**  **[1.04-1.26]** | **.008** | **0.09 (0.04)** | **1.09**  **[1.01-1.18]** | **.028** | **0.12 (0.05)** | **1.13**  **[1.02-1.24]** | **.020** |  |
| Emotional problems (SDQ) | 0.07 (0.07) | 1.07  [0.93-1.24] | .337 | 0.14 (0.09) | 1.15  [0.97-1.36] | .110 | 0.05 (0.07) | 1.06  [0.91-1.22] | .464 | 0.15 (0.09) | 1.16  [0.98-1.38] | .087 |  |
| Parental depressive symptoms (PHQ-8) | 0.01 (0.03) | 1.01  [0.96-1.07] | .614 | -0.01 (0.03) | 0.99  [0.93-1.05] | .777 | 0.01 (0.03) | 1.01  [0.96-1.07] | .682 | -0.01 (0.03) | 0.99  [0.94-1.06] | .836 |  |
| Family climate (FCS) | -0.11 (0.06) | 0.9  [0.80-1.01] | .081 | 0.02 (0.08) | 1.02  [0.88-1.19] | .787 | -0.08 (0.06) | 0.93  [0.82-1.05] | .231 | -0.01 (0.08) | 0.99  [0.85-1.16] | .913 |  |
| Personal resources (PRS) | 0.04 (0.05) | 1.04  [0.93-1.15] | .514 | -0.06 (0.06) | 0.94  [0.83-1.07] | .349 | 0.02 (0.06) | 1.02  [0.91-1.14] | .699 | -0.05 (0.06) | 0.95  [0.84-1.08] | .465 |  |
| Social support (SSS) | 0.03 (0.05) | 1.03  [0.93-1.15] | .549 | -0.01 (0.06) | 0.99  [0.88-1.10] | .806 | 0.04 (0.05) | 1.04  [0.93-1.15] | .518 | -0.002 (0.06) | 1.00  [0.89-1.12] | .976 |  |
| **Pandemic factors** |  |  |  |  |  |  |  |  |  |  |  |  |  |
| Increased digital medis use^c^ |  |  |  |  |  |  | 0.18 (0.28) | 1.20  [0.69-2.09] | .526 | 0.03 (0.28) | 1.03  [0.59-1.8] | .910 |  |
| Increased family conflicts^d^ |  |  |  |  |  |  | 0.55 (0.30) | 1.74  [0.96-3.15] | .068 | -0.31 (0.37) | 0.73  [0.36-1.50] | .399 |  |
| High pandemic burden^e^ |  |  |  |  |  |  | 0.09 (0.41) | 1.10  [0.50-2.44] | .818 | 0.49 (0.50) | 1.64  [0.62-4.33] | .318 |  |
| Loneliness (UCLA) |  |  |  |  |  |  | -0.05 (0.05) | 0.95  [0.87-1.04] | .247 | 0.03 (0.04) | 1.03  [0.95-1.13] | .436 |  |
| **Model fit** | χ²(9) = 58.05, *p* <.001  *Nagelkerke R²* = .186 | | | χ²(9) = 52.64, *p* <.001  *Nagelkerke R²* = .173 | | | χ²(13) = 63.34, *p* <.001  *Nagelkerke R²* = .202 | | | χ²(13) = 55.05, *p* <.001  *Nagelkerke R²* = .181 | | | |

*Notes.* Outcome: Eating disorder symptomatology according to SCOFF; significant predictors (*p* <.05) are indicated in boldface. Reference category: ^a^ Not female; ^b^ No migration background; ^c^ No increase in DMU; ^d^ No increase in conflicts; ^e^ Not burdened by the pandemic. Abbreviations: *CI* = 95% Confidence Interval; CES-DC = Center for Epidemiological Studies Depression Scale; DMU = Digital Media Use; FCS = Family Climate Scale; *OR* = Odds Ratio; PHQ-8 = Patient Health Questionnaire; PRS = Personal Resources Scale; SCARED = Screen for Child Anxiety Related Disorders; SDQ = Strengths and Difficulties Questionnaire; SSS = Social Support Scale; UCLA = Los Angeles Loneliness Scale.
